# Studies on Possible Ion-Confinement in Nanopore for Enhanced Supercapacitor Performance in 4V EMIBF_4_ Ionic Liquids

**DOI:** 10.3390/nano9121664

**Published:** 2019-11-22

**Authors:** Jie Deng, Jing Li, Zhe Xiao, Shuang Song, Luming Li

**Affiliations:** 1 College of Pharmacy and Biological Engineering, Chengdu University, Chengdu 610106, China; dengjie@cdu.edu.cn; 2 Department of Chemical Engineering, Sichuan University, Chengdu 610065, China; jingli0726@g.ucla.edu (J.L.); 2017226220007@stu.scu.edu.cn (Z.X.); 2016323050027@stu.scu.edu.cn (S.S.); 3 Institute of Advanced Study, Chengdu University, Chengdu 610106, China

**Keywords:** supercapacitor, porous carbon, ionic liquid, confinement effect, coulombic ordering, high energy density

## Abstract

Supercapacitors have the rapid charge/discharge kinetics and long stability in comparison with various batteries yet undergo low energy density. Theoretically, square dependence of energy density upon voltage reveals a fruitful but challenging engineering tenet to address this long-standing problem by keeping a large voltage window in the compositionally/structurally fine-tuned electrode/electrolyte systems. Inspired by this, a facile salt-templating enables hierarchically porous biochars for supercapacitors filled by the high-voltage ionic liquids (ILs). Resultant nanostructures possess a coherent/interpenetrated framework of curved atom-thick sidewalls of 0.8-/1.5-nanometer pores to reconcile the pore-size-dependent adlayer structures of ILs in nanopores. Surprisingly, this narrow dual-model pore matches ionic radii of selected ILs to accommodate ions by unique coupled nano-/bi-layer nanoconfinements, augmenting the degree of confinement (DoC). The high DoC efficiently undermines the coulombic ordering networks and induces the local conformational oscillations, thus triggering an anomalous but robust charge separation. This novel bi-/mono-layer nanoconfinement combination mediates harmful overscreening/overcrowding effects to reinforce ion-partitioning, mitigating long-lasting conflicts of power/energy densities. This interesting result differs from a long-held viewpoint regarding the sieving effect that ion-in-pore capacitance peaks only if pore size critically approaches the ion dimension. Optimal biocarbon finally presents a very high/stable operational voltage up to 4 V and specific energy/power rating (88.3 Wh kg^−1^ at 1 kW kg^−1^, 47.7 Wh kg^−1^ albeit at a high battery-accessible specific power density of 20 kW kg^−1^), overwhelmingly outperforming most hitherto-reported supercapacitors and some batteries. Such attractive charge storage level can preliminarily elucidate an alternative form of a super-ionic-state high-energy storage linked with both the coordination number and coulombic periodism of the few ion-sized mesopores inside carbon electrodes, escalating supercapacitors into a novel criterion of charge delivery.

## 1. Introduction

Prompt charge/discharge kinetics, prodigious longevity, prominent power density, ultralight weight, pronounced safety and so on, these dominant advantages relative to various batteries have brought to the fore the supercapacitors (SCs) in academic and industrial sectors [1]. Particularly, the ones assembled from carbon materials can take full advantage of high electric conductivity, low cost, elementally/organizationally tunable properties, eco-compatibility, rich porosity and large specific surface areas [1]. These high-profile carbon-based SCs can store/deliver energy by the reversible adsorption/desorption of oppositely charged electrolyte ions across electrical double layers (EDL) in the vicinities of electrolyte/electrode interphase boundaries. Thus, their supercapacitive behaviors positively rely upon the free surface sites easily accessed by electrolyte ions under various running conditions, while the charging and discharging rate response is absolutely controlled by the ionic motion onto and off the electrode surfaces, respectively [2]. Under this background, lots of carbon allotropes (including the carbon nanocages, onion-like carbon, carbon nanotubes, graphene, etc.) even with the huge specific surface areas above 2000 m^2^g^−1^ have been prepared to attain the respectable capacitance of 100–200 Fg^−1^ in various aqueous electrolytes [3]. Unfortunately, their specific energy densities invariably stabilize at 4–6 Wh kg^−1^ and are remarkably pale in comparison with the batteries’ counterparts of 30–150 Wh kg^−1^, thus significantly inhibiting their industrial development. Apart from a rise in the ion/electron diffusion obstacle (albeit with a slight escalation in the rate loadings), the major reasons behind such low specific energy densities come from a disincentive that the inevitable water splitting reaction fiercely emerges and abruptly exacerbates as soon as the voltage windows surpass some certain low value [3,4]. Thus, a pressing technological stipulation of the ever-faster and steadier charge storage capability during the momentary charging process is needed. There must be some increasing attempts into a new strategy to fulfill the extraordinary energy density similar to the batteries while not prejudicing the typical virtues (power density, lifespan, etc.) of the SCs. 

In theory, such appealing integration of the exceptional energy density with salient power density demands both a nontrivial substance, which is capable of preserving plentiful charges (like ionic liquids, ILs) and an ingenious morphostructure that instantaneously enables transporting abundant charge carriers (electron/ions) at the fixed charging/discharging time. Since the energy density (E) is essentially determined by the voltage window (V) utilized for SCs according to the equation of *E* = 0.5 *CV*^2^, such performance could easily come true for an optimal system that includes the robust electrolytes with the considerably superior electrochemical window/stability to the well-established solvent-based systems [1,2,3]. Fortunately, ILs, a new kind of low temperature molten salt (also called coulomb liquids), has distinguished themselves as the most prospective candidates for the well-defined aqueous solutions. ILs are comprised of the identical quantities of the bulky organic cations and organic (or inorganic) anions of unequal sizes. Such a chemical nature leads to unparalleled traits in terms of the expanded operating potentials (in excess of 3.5 V), non-ignitability, large cell working temperature scope (−50 °C–150 °C), negligible evaporability, a sumless combination of the varied cations/anions [5,6]. Frustratingly, the perfect ILs/electrode combinations are hardly achieved for the well-structured carbon materials reported so far due to some intrinsic attributes, such as the great viscosity, large ion radius, minuscule ionic conductivity of solely ~2 mS cm^−1^ vs. 1 S cm^−1^ of aqueous environments, strong electrostatic coupling between ions, and breakdown of a standard mean-field response. In comparison with aqueous systems, such intrinsic attributes can unavoidably lead to the more intricacy not only of the structural heterogeneity and microphase separation at the nanometer scale but also of the consequent ion electro-sorption and charge storage mode in the nanoporous carbons [7]. For example, charging takes place via co-ion desorption, counter-ion adsorption, co-ion-counter-ion swapping or their random combinations, wherein co-ions and counter-ions refer to bearing the same or opposite charge to the electrode surfaces at which they are situated. In the meanwhile, these cations and anions of ILs are characterized by the obviously asymmetric chemical structures, and thus the positive and negative electrodes have the operating voltage maldistribution and volatile electrochemical functions [2,7]. Ultimately, this salient difference renders maladaptive the manipulation rationales (which have been well established previously in aqueous electrolytes) when they are mechanically translated into ILs. Such marked lacking in precisely delineating the behavior-structure-parameter correlation can severely impede a tempo forward towards upgrading the relevant carbon materials for the realistically large-scale application. Resultantly, the energy/power delivery rating performance of the current carbon/ILs systems scarcely exceeds the cases for contemporary Li-ion cells. As such, it is imperative to overcome such disadvantages (the extremely poor ILs/carbon compatibility) through collectively exploring a flexible fabrication method for the highly electroactive carbon electrode materials, which will have the fast transport kinetics and optimized surface utilization efficiency at broadened current rates.

By and large, maintaining the exceptional capacitance and rate loading within the carefully shortlisted high voltage ILs electrolytes (such as EMIBF_4_ > 3.5 V) requires the sufficient charge carrier fluxes fluently through the tortuous tunnels of the carbon materials against the appreciable steric and electrostatic side-effects (from certain interactions of ion-pore walls, intra-ions and inter-ions). An emerging dilemma looms large that the ILs electrolytes tend to pose a rather lagging charging dynamics and thus reducing the SCs’ power densities, because the ion transfer is sluggish in the bulk phases and can be even tardier inside the nanopores. The up-to-date theoretical investigations aiming at the precise impacts of the carbon’ structures upon SCs have unveiled that the in-nanopore self-diffusion and -exchange process of ion currents turns highly pivotal. A nomenclature of the degree of confinement (DoC) has been coined as a central benchmark with which to quantify the locally scaled perturbations of both the ionic population and interionic configurations around the electrified interfaces under nanoscaled confinement [8]. Put another way, DoC can straight measure a spatial microenvironment (the local position, etc.) where ions are restricted to figuratively characterize how they “see/react” to complex carbon pore structures. The high DoC level notably attenuates and even eliminates the mighty inter-molecular attractions amidst hithermost neighbors so as to engender an anomalous superionic state of ions. The superionic state of ion is a much-anticipated possibility, which not only disarranges the ILs’ bulk structural motifs (of each central ion being encircled immediately by the successive ion coordination shell of the alternating opposite charge), but also changes the electrical neutrality of the ILs’ bulks. In this case, supplanting a positive ion (within a densely packed coordination envelope intimately along the overall perimeters of a central negative ion) with another negative ion can turn into an energetically amenable action [9,10]. In essence, DoC of ions within the carbon nanopores represents a comprehensive structural descriptor towards recapitulating the ease with which a variety of the related electrochemical events sustainably occur inside nanoscaled pores of carbon electrodes under a collective effect (which all sorts of factors—such as the pore ionophobility or ionophility, breadth, shape, curvature, conductivity, sinuosity, functionality and stiffness—exert upon the ion packing configuration and morphology shift in nanospaces). Thus, for the ILs-wetted carbon SCs, the ion self-diffusion constraint does matter much in that the slow charging dynamics will predispose the capacitance to slump due to the markedly increased overpotentials [11]. Such preoccupations clearly denote that the carbon nanopore’ traits that can condition the final DoC level and thus be indispensable to achieve a good energy-power handling are the underpinnings but difficulties of updating the ILs-based carbon SCs into an uppermost echelon among various nascent energy storage devices. Nevertheless, in the case of the mainstream carbon allotropes involving the activated carbon, graphene, activated graphene and even ordered porous carbons, it remains very tough to afford an accurately-regulated pore property especially with a perfectly controlled pore size distribution (PSD). The ubiquitous ultra-narrow pores in the synthetic carbons can usually impair the ion conveyance/partitioning [1,2,3,4,12]. Therefore, the carbon material design norm built upon the present insights turns out rather scant for making the SCs’ energy densities approach the batteries while not reducing the output power.

Herein, we heuristically show the construction and nanoconfinement functions of ionic species from ILs within a three-dimensional porous carbon structure. The tailor-made electrode materials with desirable textural parameters (well-defined pore width dispersion, enriched small mesopores, large specific surface area, adjustable pore architecture, N-self doping and monolithic macroporous network) are derived through a facile, eco-friendly and costless salt-templating tactic based upon a KNO_3_-crosslinked gelatin biopolymer aerogel. The effect of in-nanopore confinement upon the ILs’ charge storage in these synthesized samples is deciphered. All consistent data clearly reveal that the pore topology characteristic effect of the as-prepared nanocarbon initiates a hallmark of the “micro-environmentally nanoconfined” preferable ion reconfiguring/distribution, which can conclusively dictate the quantity/mode of the adsorbed charged ions and thus the capacitance. The high DoC, resulting from a perfectly-modulated narrow PSD of the single and double ion-sized nanopores, can essentially break the coulombic symmetry criterion of the bulk ILs and catalyze the occurrence of the possible ion conformation/phase perturbations in the course of charging/discharging. The mighty screening of interionic electrostatic correlation by image charges triggered around highly confining sites guarantees the production of non-coulombic ordering structures. Such countervailing response to the non-uniform electrostriction combined with an extremely constructive EDL interference elicits a “micro-environmentally confined” favorable interionic restructuring. This restructuring effect can amazingly deflect the electricity storage rules from the well-established EDL compression theory and can well account for an atypical increase in the experimentally gauged capacitance in our deliberately designed carbon nanopores [9,10,13,14]. Moreover, a highly interlinked monolithic graphitizable framework boosts the superior electronic conductance function, while its graded porous texture, well conjoining the small-scaled macropores within the 3D skeleton and regulatable micro-/meso-holes in the carbon matrix, fosters the fluent mass diffusion and shuns the transmission restrictions across the total electrode configurations. This unique morphostructure can result in the interconnecting charge carrier conveyance shortcuts, which can ensure the good capacitive properties even under the rapid charge/discharge speeds at a high weight loading density.

## 2. Materials and Methods 

The targeted electrode materials were prepared with a green and facile salt-template way, and their structural property was characterized by scanning electron microscope (SEM), transmission electron microscope (TEM), X-ray photoelectron spectroscopy (XPS), N_2_-physisoeprion and so on. Electrochemical behaviors were assessed by cyclic voltammetry (CV), chronopotentionmetry (CP) and electrochemical impedance spectroscopy (EIS). All details are provided in supplementary materials.

## 3. Results

### 3.1. Materials Synthesis and Characterization

The KNO_3_ salt is carefully singled out as an outstanding sequential template for evaluating its catalytic graphization and topotactic conversion effects. The flexible hydrogel–aerogel method based on the gelatin biopolymer biomass has found wide applications to various fields because of the cost competitiveness, full supply, plentiful multifunctional groups, non-pollution, excellent solubility, chelation, gelling, emulsifying and foaming. However, this strategy has rarely been used to synthesize the multi-purpose nanocarbons employed for electrochemical energy storage/conversion areas [15]. In this sense, the aqueously soluble and neutral KNO_3_ displays the excellent bio-compatibility with gelatin because totally ionized species of KNO_3_ in water does not alter solution’s acidity and alkalinity so as to thoroughly inhibit the adverse proteolysis of gelatin in the non-neutral environments. Thus, the good bio-compatibility can permit not only the long-term survival of an ion-homogeneously complexed hydrogel but also its subsequent successful recrystallization into 3D salt-gelatin adducts. Such salt–gelatin compounds can further transform into the diverse derivative crystallites with the amorphous carbons closely adjoining the self-supporting grains so as to exert the varied roles on the carbon growth mechanisms. The final pseudomorphic evolution trend of carbon sources in oxygen-lean pyrogenic circumstances is mainly dictated by the thermal annealing conditions and salt/gelatin dosage ratios, e.g., with an elevated carbonization temperature driving the pseudographitic array together with the augmenting crystallinity, dilating inter-graphene spacing and grading porosity, vice versa. As such, the excellent inter-crosslinking mechanism (due to the strong bonding between ligands of gelatin and KNO_3_ salt) can in situ disperse ions on molecular levels, evenly immobilize ions within whole gelatin gel scaffolds and finally unleash huge chances of simultaneously achieving catalytic graphitization and sequential templating to procure the expected nanocarbons with both the finely-controllable multi-porosity and charge conductivity. Multi-porosity and charge conductivity are seen as two leading figures-of-merit to fulfill the high confinement extent and efficient charge storage/delivery across overall intra-electrode substance interphases. The nanocarbons’ electricity storage process nearly rests with a much quicker surface-constrained kinetics (than can induce the unusually superior rate handling) instead of a comparatively tardy semi-infinite diffusion pattern (as arises in the bulks of battery/pseudocapacitance-type materials) [1,2]. Nonetheless, owing to the high susceptibility of activated carbons to poor surface accessibility and steric osmosis, only in the finely-controlled porous nanocarbons or at a rather small rate loading can the exceptional charge storage with both the generous quantities and rapid velocities be easily accomplished in spite of the extensive attempts at both the nanotextured engineering and at the utilization of the carbon-based hybrid nanoarchitectures. In conventional porous carbon electrodes, the whole charging abilities are foiled by the deficient channelling/sampling of ions to the electrode substance surfaces [1,2,3,4]. All taken together, the acute response of the superb energy storage behavior of nanocarbons to confinement effects qualifies a retrofitted coordinative KNO_3_-based gelatin sol–gel methodology as an elastic arena to delve into the rational design of the ILs-compatible carbon nanomaterials and their charge storage mode/kinetics.

**Figure 1:**
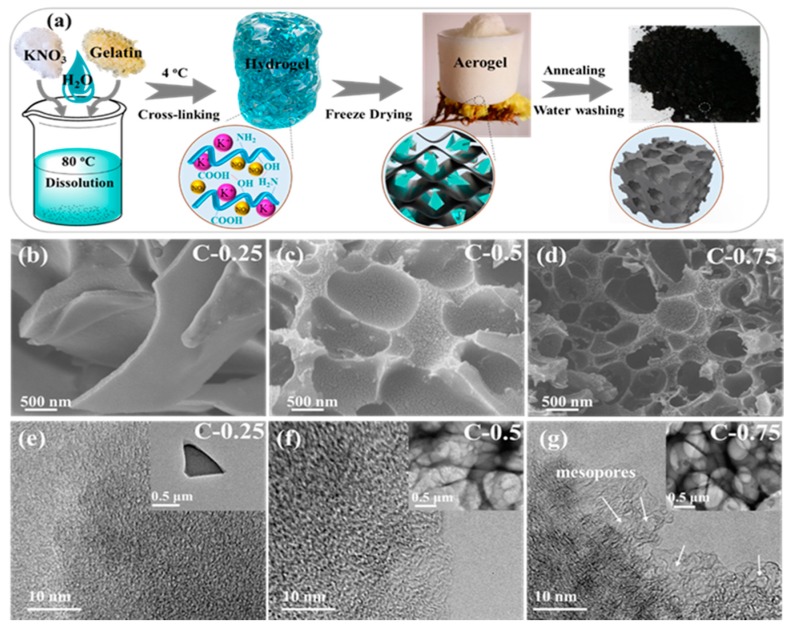
(**a**) The synthesis scheme of the gelatin-derived porous carbon by KNO_3_-confined pyrolysis strategy. (**b**–**d**) SEM and (**e**–**g**) TEM images.

**Figure 2:**
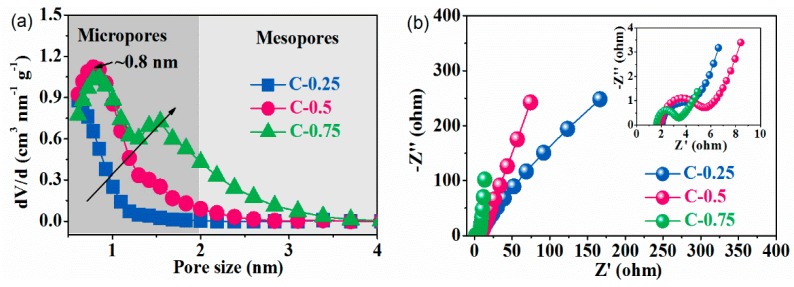
(**a**) Pore size distribution from N_2_ volumetry and (**b**) Nyquist plots from the electrochemical impedance spectroscopy (EIS) spectra (measured in the frequency range of 10^5^–0.01 Hz).

Expected nanocarbons were derived from a stepwise procedure in which the monolithic carbon networks first united uniformly with the KNO_3_ nanocrystallites and then transformed into the constituently/configurationally hierarchical products (Figure 1). Final multiporous superstructure is designed to wet using ILs and hence improve charge storage. The reparation procedures as shown in supplementary materials start with the self-sacrificing KNO_3_ nano-crystal co-porogen-catalyst-etchant. The KNO_3_ nanocrystals are self-organized and homogeneously confined in the continuous protective carbon framework just only by means of polymerizing an aqueous solution of KNO_3_ and gelatin into a salt nanocrystallite-inserted spongy xerogel. An atomically dispersive hydrogel system incipiently forms on account of the strong coordination of the protein polymer chains with the salt’s ion during concentration (Figure 1a). Next, this hydrogel system turns into a self-cross-linked salt-biopolymer clathrate hydrogel by the hydrogen bonding when cooling down (Figure 1b). Eventually, a foam-like gelatin biopolymer aerogel is induced with the abundant salt nanofillers as endotemplates (uniformly interspersed in situ amidst scaffolds) as a result of an overall dissociation-recrystallization-nanospace occupation evolution mechanism driven by the KNO_3_ salts (Figure 1c) [16]. During the following elevated-temperature pyrolysis, such equally embedded KNO_3_ nanofillers can withstand a structural disintegration below 600 °C to notably stabilize the 3D coherent carbonaceous framework, and afterwards commence to smoothly decompose into the K_2_O solid products (which firmly accrete on the carbon scaffold surfaces) and a variety of the pyrolytic gases (O_2_, N_2_ and NO) under the higher temperatures [16]. After the complete vanishing of the nanospatial occupation by fillers, a large number of the equivalently shaped and sized macropores to the parent KNO_3_ fillers are topotactically mirrored/transcribed throughout the carbon scaffolds, yielding a 3D honeycomb-like macroporous superstructure with an extraordinary dimensional interconnectivity and continuity (Figure 1b,d). The population, width, and connectedness of these conformally duplicated vacancies can dramatically vary with the salt dosage, e.g., a greater salt amounts, a smaller macropore size, and a higher connectivity (Figure 1b–g). During the salts’ in situ decomposition, the highly corrosive K_2_O solid product along with the afterwards-formed CO_2_ will be fiercely reduced by the proximal carbons (K_2_O + C → K + CO_2_, CO_2_ + C → 2 CO) [17], simultaneously accompanied by two paragenetic effects of the in situ etching away of carbon lattices into massive micropores and of massive heat emissions. These pyrolytic gases (N_2_, O_2_, NO and CO) can synchronically expand the width of these micropores into small mesopore regimes (Figure 1e–g), while the released heats induce a very high-temperature zone localized at peripheries of such newly-minted pores to improve the aromatization/exfoliation of bulk carbon layers by K. This highly integrated etching-expansion-graphitization-delamination mechanism tends to occur at around the chemically more active O/N defective centers and extends along the basal planes of carbon lattices. As a result, the growingly denser, more and broader pore microstructures with the cylindrical-like shape, high curvature, satisfactory heteroatom doping and dominantly atom-thick sidewalls are created when the salt dosage increases from 0.25 to 0.75, as explicitly evidenced by N_2_ porosimetry (Figure S1), XPS (Figure S2) and TEM (Figure 1b–g) results. Finally, a simple/non-destructive water washing procedure can totally remove the K residues, giving rise to the structurally/compositionally refined nanocarbon with the high yield and purity. The pith of this effective strategy lies in the in situ-intercalated KNO_3_ salt crystals, which simultaneously exert multifaceted implications upon the allostery of the gelatin biopolymer during thermal treatment. This sequential templating effect on the final morphology-structure of the targeted carbon products highly depends upon the salt dosage. Using a various salt/gelatin ratio, a perfectly controlled pore-width-distribution narrow pore nanostructure of the order of ion dimension is achieved to derive three distinct versions of the designer nanocarbons: (1) the purely ultramicro-porous carbon (C-0.25) with the pore size being around 0.5 nm, (2) the predominantly microporous carbon (C-0.5) with the pore diameter approximating 0.8 nm and (3) the equally micro-/meso-porous carbon (C-0.75) with the pore breadth distribution primarily being around 0.8 nm and 1.5 nm, as seen in Figure 2a. Their detailed structural information is given in Table 1.

### 3.2. The Electrochemical Test

To analyze the impact of the configurational properties of carbon upon the charge transport dynamics, electrochemical impedance spectroscopy (EIS) was executed in advance to obtain the exact clues regarding the electrode/electrolyte interphases and electrokinetic traits. As shown in Figure 2b, all curves featured three different domains, a small quasi-semicircle in high frequency region followed by a pseudo-straight line at low frequency region and a medium-frequency transition slope segment between 5 and 100 Hz. Such traits signify a non-faradaic phenomenon, which an equivalent circuit can further evidence [18]. At low frequencies, the straight line was virtually upright for C-0.75 but was notably inclined on C-0.5 and particularly C-0.25, validating that C-0.75 behaved purely capacitor-like with ultra-rapid construction of EDLs. Even though there was enough time for ions to approximate a steady status, most of micropores in C-0.25 remained still unavailable by ions so as to deviate from the ideal capacitive response. Through extrapolating vertical portions of these inclined direct lines onto a real axis, the internal equivalent series resistance (R_ESR_) was gauged to be roughly 5.73, 4.28 and 3.41 Ω for C-0.5, C-0.25 and C-0.75, respectively. The lowest R_ESR_ of C-0.75 agreed with its fairly quasi-symmetric GCD curve, near-box CV profile, and much restricted IR_drop_. The lowest R_ESR_ of C-0.75 most probably ensued from the concerted combination of the multifaceted causations: e.g., (1) the local rise of in-pore migration incurred by impingement upon stiff channel sidewalls [19]; (2) low tortuosity related with such less turbostratic pore arrangement in carbon backbone that ions percolate in a direct way [20]; (3) a short-length mass diffusion pathway stemming from interpenetrated multi-porosity [16]; (4) the shortened transportation interfaces owing to the closer ion attraction to walls [21] and (5) conformational metamorphosis of electrolyte ions in the nanopores heterogeneous to the neat bulk electrolytes, as evidenced by some studies that the carbon-ion interplay is able to profoundly affect the molecular structure of nanopore-confined ILs [22,23,24,25]. R_ESR_ of a capacitor finally shapes its frequency response and rate; a smallest R_ESR_ of C-0.75 here explains its most conducting carbon network very conductive to storing/releasing adequate energies. In elevated frequencies, the system performs like a resistor as the electrolyte ions cannot circulate sufficiently [26]. The mid-frequency transition part, also termed as a ≈45^o^ straight line, could reference the frequency sensitivity of the ion spread within the electrolyte-wetted porous organization. Its projection on real axis allows access to ion diffusion impedance (R_ion_), a critical figure-of-merit to symbolize the rate/power ability in the course of charging/discharging [27]. The 45° sloped line was extremely short for C-0.75 but very long for C-0.25, illustrating that the electrolyte ions could easily permeate and go through the internal porous frameworks of C-0.75 with the shortest ion diffusion expressways and fastest ion diffusion rate, expediting the electrochemical processes. Quantitatively, the progressive variation of the projected distance along x-axis on three electrodes marks a consecutive diminishment of R_ion_ from 0.92 Ω for C-0.25 to 1.41 Ω for C-0.5 and 0.38 Ω for C-0.75, with the rising pore size and mesopore volumes in carbon skeletons. The absence or severe depression of the half-loop within the immediate frequency unravels a trivial charge transfer resistance (R_c_) around electrode/electrolyte interfaces due to a close proximity of ions to the pore sidewalls that significantly change the local micro-environment where ions are located [28,29,30,31]. In the insert in Figure 2b, the semicircle radius was greatly smallest for C-0.75 but biggest for C-0.5, manifesting the decrease of charge transfer resistance for dual-mode pores but an increase of charge transfer resistance for ion-sized pores. Quantitatively, the significantly increased R_c_ from 1.7 Ω on C-0.75 to 2.3 Ω for C-0.25 and 3.7 Ω for C-0.5 indicates that the electrode/electrolyte interface areas were poles apart across the electrodes. The R_c_ evolution is consistent with the R_ESR_ variation. The R_c_ difference must correlate with the varied microstructure of EDL from sample to sample due to both the pore structures and to the electron conduction resistance of materials. Overall, the foregoing outcomes unveil that the diffusion dynamics of ILs could be wisely reinforced through manipulating the pore structures of the salt-templated nanocarbons. The improved in-nanopore ion relocation and movement must closely be relevant to both an enhanced electrokinetic signature and an uncommon charge storage mode of ILs in the unique pore architectures.

**Figure 3:**
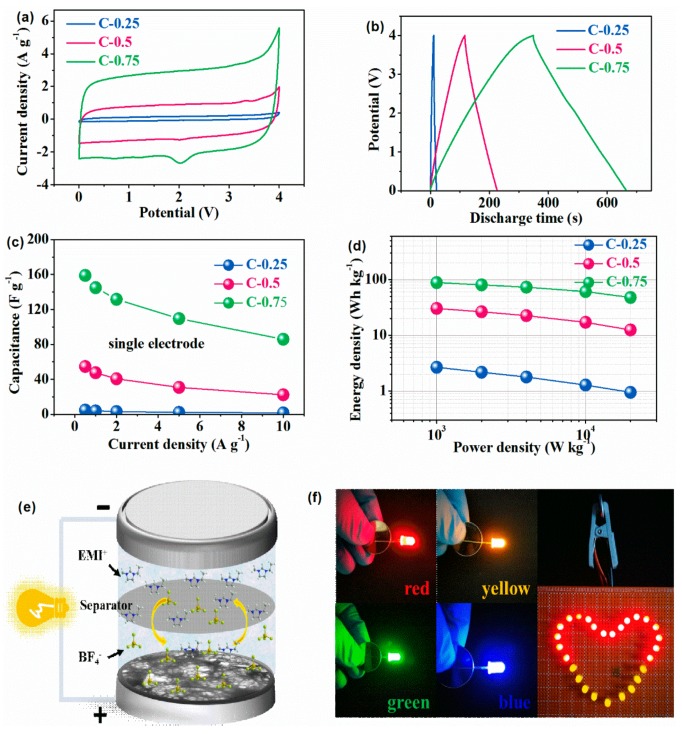
Electrochemical performance of two-electrode symmetric supercapacitors (SCs) in (a–c) EMIBF_4_ ionic liquids (ILs). (**a**) Cyclic voltammetry (CV) curves conducted at 20 mVs^−1^, (**b**) chronopotentionmetry (CP) curves at 0.5 Ag^−1^ and (**c**) rate capability. (**d**) Ragone plot. (**e**) Illustration of the assembled 4 V coin-cell using C-0.75 as electrode materials. (**f**) Its practical demonstrations in lighting LEDs with different colors/working voltages or in parallel.

The probable electrochemical processes in ILs (EMIBF_4_) were further scrutinized by means of the chronopotentionmetry (CP) plus cyclic voltammetry (CV) tests. Miraculously, a working voltage window can be consistently stabilized at as high as 4V without noticeable polarization. Universally, CV plots (Figure 3a) in a slow state of 20 mVs^−1^ describe the very quasi-orthogons, typical of an ideal efficient EDL capacitor. Unexpectedly, both a narrow hump nearby a cell voltage of ≈2 V and a more protrusive and wider bulge between 3 and 4 V are rather conspicuous on C-0.75, very weak for C-0.5, but practically invisible over C-0.25. The marked differences at high potentials mean occurrence of other distinct charge storage mechanisms and ion transfer properties over varied electrodes. It is well acknowledged that the higher nitrogen doping level, the larger pseudocapacitance contribution to the overall capacitance. The obvious shape distortions or additional peaks of CV usually occur at a quite large nitrogen doping level (>5%), while the redox peaks due to the nitrogen-including groups have proven to normally emerge under a very low potential range (<2 V). Interestingly, the intensity enhancement sequence of such peaks rigorously consists with the order that the alien dopant fraction dwindles from a significant level of 9.45% in C-0.25 down to a trace content of 1.68% for C-0.75. Thus, these results can safely rule out a predominant contribution of pseudocapacitance effects originating from the functionalities’ Faradaic redox reactions, and can, meanwhile, strongly substantiate the fact that a biggest structural disparity (namely a pore size dispersivity) holds fundamentally responsible for the additional electrochemical processes. The precise role of such dopants on the carbon surfaces in modulating the capacitance in ILs is out of the scope of our study. According to the literatures, the dopant most possibly plays a main role in enhancing the wetting of carbon surfaces by ILs. The above appearance and symmetry identities can still get well preserved while the sweep speed jumps up to 200 mVs^−1^ (Figure S3). The current density or the amplitude of variation in CV absorbance is linearly dependent on the scan rate with the low resistive contribution because more ILs’ ions enter pores to compensate for the applied voltages. Instead, the related CV patterns of other two electrodes (Figure S3) suffer from an increasingly distorted, asymmetric and fusiform appearance typical of the sieving effects because ions cannot sample the pore surfaces rapidly enough to counterpoise the changing voltages [32]. This clearly evidences that EDLs integrated with the newly introduced electrochemical processes have the fastest charge/discharge kinetics for C-0.75, in agreement with the EIS results.

Consistent with CV data, all CP data depict near-isosceles triangles with both pretty inclined slope and limited polarization-initiated voltage drops (IR_drop_; Figure S4). Relative to the dominantly micro-porous and totally ultra-microporous samples without finely-regulated nanopores (C-0.25 and C-0.5), a hierarchical micro-structure (C-0.75) having the subtly modulated nanopores persistently affords more attractive specific capacity combined with a lower IR_drop_ and capacity decay under a set current density. Such disparity growingly heightens with a mounting current loading. Concretely, an anomalous specific capacitance no less than 158.9 F g^−1^ under a current density of 0.5 A g^−1^ is acquired for C-0.75, as opposed to the poor levels of 54.7 F g^−1^ for C-0.5 and 4.8 F g^−1^ for C-0.25, a dramatic increase of three times and more than one order of magnitude, respectively. Over a broad scope of current densities from 0.5 to 10 A g^−1^ (Figure 3c), in spite of continuous capacitance deterioration for all electrodes, C-0.75 can always go on offering a well-behaving CP plot and outstanding capacitance retention. The capacitance of C-0.75 smoothly falls above 5 A g^−1^ and ultimately accomplishes 85.9 F g^−1^ (54.0% of initial value) at 10 A g^−1^, which outnumbers the negligible values of 1.7 F g^−1^ (35.0%) of C-0.25 and 22.4 F g^−1^ (41.0%) of C-0.5 and vies against most of the top-level carbon materials in ILs recorded so far [3,4]. A very high/stable coulombic efficiency up to 98% again underlines inexistence of the prominent Faradaic redox reactions in selected potential scopes [33]. A notable variation in the rate capabilities represents a noted enhancement with the increasing nanoporosity [34]. Given the fact that C-0.25 with a high nitrogen doping level of 9.45% delivers a trivial capacitance of 4.8 F g^−1^ while C-0.7 with the trace nitrogen dopant content of 1.68% gives an excellent capacitance of 158.9 F g^−1^, it should be stressed again that the other factor rather than the doping underlies the substantial capacitance improvement in our case. Most significantly, the Ragone plot (Figure 3d) shows a rather parallel line plot to the *x*-coordinate when increasing from 1 kW kg^−1^ up to a very high specific power density of 20 kW kg^−1^, indicative of a superior energy/power rating. A specific energy density as large as ~88.3 Wh kg^−1^ can be obtained at a specific power density of 1 kW kg^−1^ and still remain around 47.7 Wh kg^−1^ even at 20 kW kg^−1^ for C-0.75, higher than 30.4 Wh kg^−1^ on C-0.5 and 2.7 Wh kg^−1^ on C-0.25 at 1 kW kg^−1^. Indeed, the specific energy density reaches the purview of high values among most of the ever-reported carbon materials, and even one order of magnitude exceeds that of commercial EDL capacitors without impairing the appealing power handling capabilities. To demonstrate the lifetime, a 5000 cycles consecutive charging/discharging was implemented at a very high current density of 10 A g^−1^. A recommendable capacitance retention of 83.3% (only 0.33% decay per cycle) is attained for C-0.75 (Figure S5), which is comparable to most of reported carbon/ILs system (as compared in Table S1). To exhibit the practically related applications of the materials, a 4 V two-electrode coin-cell was configured as demonstrated in Figure 3e,f. It could power LEDs with different colors (red, yellow, green and blue) between the running voltages of 1.8–3.5 V. Besides, one coin cell could directly light 27 LEDs in parallel with a heart-shaped pattern. For clarity and brevity, all key parameters of the SCs after and before optimization have been compiled in Table 2.

## 4. Discussion

Such impressive capacitive function must derive from optimal pore structure effects in terms of mixed micro-/meso-pores as well as the occurrence of a unique fashion of electrochemical processes. This particular electrochemical events must arise within volumes of ILs in place of merely the surface EDLs condensations, as will be fully discussed below. Surprisingly, the purely ultra-microporous C-0.25 has the empirically recommendable textural parameters with both sizable specific surface area (1084.3 m^2^ g^−1^) and pore volume (0.402 cm^3^ g^−1^), but it is nearly inert to store energies. In sharp contrast, an only 2–2.5 times increase in the specific surface areas (2744.6 m^2^ g^−1^) and pore volumes (0.826 cm^3^ g^−1^) can incredibly lead to a 33 folds capacitance boost. This sheer anomaly seems very appealing but counterintuitive, severely deviating from the opposite manner that the capacitance typically behaves for the well identified aqueous electrolytes [1,2,3,4]. It doubtless manifests that the tailored nanoporosity (rather than both “incessantly” sought specific surface area and heteroatom functionalization) acts as an underlying prerequisite to sustain an efficient charge storage in the ILs-soaked carbon SCs through a special working mechanism. The notably fluctuating capacitance with the pore structure effects can hint that the charging mechanism around differently confined interfaces owns the distinct signatures across samples. Succinctly, the EDL capacities inherently unveil the degree to which the electrostatic potentials around interphases can get countervailed by the ion adsorbents, which regularly aggregate there. For example, a superior compensation, a larger capacitance. More importantly, the electrostatic potentials can govern the total thickness (Debye length) of alternating co-ion/counter-ion layers. The representative width of every EDL layer equals the magnitude of an ionic dimension, finally giving a high electrostatic potential field. Within this field, the net electrode surface charging process must be sourced in a facile ionic charge separation and its subsequent imbalanced positioning around the interface areas, and be finalized by not only innate traits of electrolyte ions (molecule construction or type) but also a fine specification of the confining states. In the restricting steric cavities, the crystal configurations cooperate with some additional phenomena (such as quantum mechanical response, symmetry-breaching factor, templating behavior, capillary effect and screening of surface charges) so that the interfacial morphology/phase properties of ILs highly hinge upon the micro-environments, for example, the externally used steric restriction and the sophisticated interatomic/intermolecular electrostatic correlations (like coulomb interaction, hydrogen bonding, polarization, self-imposed surface energy and van der Waals force) [8,9,10,22,23,24,25]. In view of this basic principle, the interfacial ion structures (including the composition, polarization, orientation, coordination and EDL micro-structures) are qualitatively explored to understand its close connection with the drastically varied EDL capacitance in the diverse nanopores, as follows.

### 4.1. The Ion Partitioning on C-0.25

**Figure 4:**
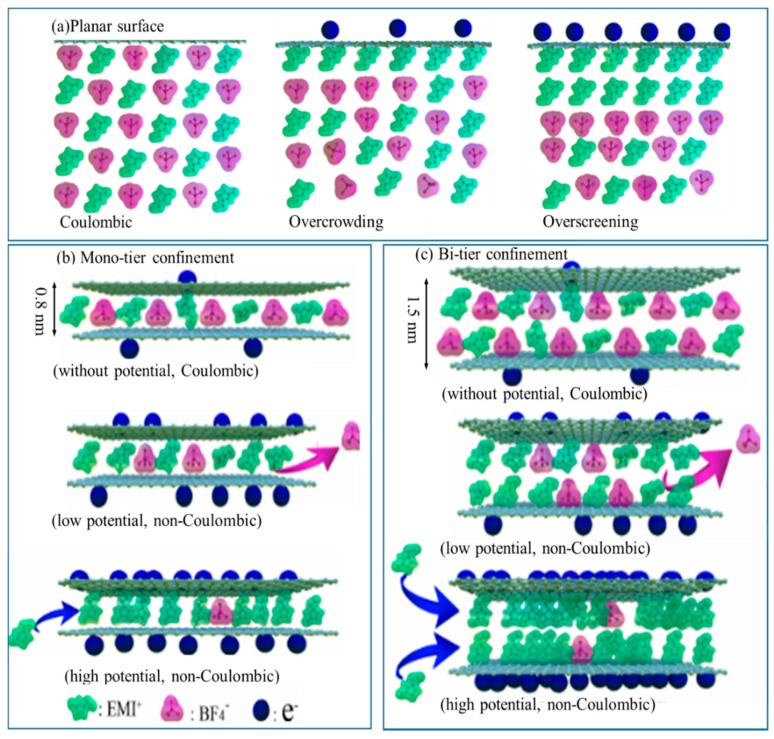
Configurational anomalies of EMIBF_4_ situated in different carbon surfaces. Notional negative electrode-electrolyte interfaces working in EMIBF_4_ restricted to standard open carbon surfaces (**a**), single ion-sized pore (**b**) and double ion-sized pore (**c**). Local ordering transitions including the perturbations of the ion coordination number and the ion phase conformations underlie the efficient energy storages.

In fact, pore width of purely ultra-microporous C-0.25 is mainly centered at about 0.5 nm, even much smaller than the crystallographic diameter of desolvated EMI^+^ ions (~0.76 nm), so its rich ultra-micropores are too narrow for EMIBF_4_ to admit into or access due to a large physical diffusion energy barrier of ions entering nanometer pores in spite of the robust driving forces from the applied potentials. It follows that an overwhelming portion of the ultramicroporous interiors/interfaces are so inaccessible as to be no longer utilized to build up abundant EDLs even during a slow discharge and charge stage at 0.5 A g^−1^. In another word, the minimal critical pore size for EMIBF_4_ to store energy via forming the efficient EDLs is 0.76 nm, with the critical energy cost for the ion’s ingress. Below this threshold, owing to a charge inversion (i.e., an oscillatory sign variation in local charge density) [5,6], the ion species from ILs have to adsorb and periodically re-orientate in the shape of a long-range ordered multilamellar (lamella-by-lamella) assembly of the alternately distributed cations and anions outside the charged ultra-microporous surfaces of C-0.25. This situation resembles the picture over the standard planar electrode surfaces. As illustrated in Figure 4a, the oppositely charged ions in this layout almost adopted the same coulomb ordering structure as IL’ bulk phase and thus had such high long-range mutuality/nexus to conjugate the polarization of the headmost adsorption tier with that of the contiguous tier. This results in the damping charge density oscillation that the first counter-ion tier can achieve a peak density while the next co-ion-rich tier owns a smaller density but still in excess of bulks [35,36,37]. That is, to satisfy the charge neutrality for the entire systems, the charges of the first adsorption tier outnumber that of the electrodes but get immediately offset by the second tier. Such compensation behavior can propagate beyond such a few tiers that solely a tiny portion of absorbates is efficiently utilized during electricity input/output. Thus, merely a trivial fraction of ion entities in EMIBF_4_ participate in the charging actions whereas a large multitude of the extra ions function like non-charged conglomerations, resembling a spectator solvent utterly extraneous to energy storage. In this case, C-0.25 with the abundant but useless ultramicropores can in reality be rationalized as the exohedral nonporous granules, along which the open charge mobility (adsorption on open surfaces over charging and departure from the open surfaces during discharging) with only an insignificant variation in bulk concentrations (Fermi-like distribution) yields a purely perm-selective mechanism. As viewed clearly, both the harmful lattice saturation/overscreening effect (Figure 4a) and the energy consumption (due to competition between the energy barrier of ions diffusing into ultramicropores and the driving force from applied potential) lastly lead C-0.25 with big surface areas of 1084.3 m^2^ g^−1^ to only store the quite small energy of 4.8 F g^−1^, in quantitative line with the stimulation-derived values on the open planar graphite [38,39,40].

### 4.2. The Ion Partitioning on C-0.5

When simply by increasing the KNO_3_-dosages the ultramicropores are enlarged to 0.8 nm in diameter, identical to size of naked EMI^+^ ions, the predominantly microporous C-0.5 can exclusively “house” a single tier of the electrosorbed ions. These electrosorbed ions can be impelled into micro-pores through a combination of external potentials, capillary forces, ion-wall electrostatic interactions, image forces and so on. After the ingress of the electrosorbed ions into the 0.8 nm pores, the molecular planes of cation and anion in the pore’s heart can primarily arrange vertically with the pore sidewalls with a quasi-packing alignment along 0.8-nm pore directions [41,42]. Resultantly, such distinct in-pore ion arrangement must suppress the so-called multi-lamellar structures as arising in C-0.25 [43,44]. In another word, the 0.8-nm micropores of C-0.5, which match the ion size in dimension can significantly change both the ion partitioning into ion-sized micropores and the interfacial properties of the ionic species. Contrary to C-0.25, C-0.5 behaves itself as a real endohedral porous structure; its Debye screening length equals its steric size. Similar to the extensively detected desolvation of the hydrated ions in small carbon nanopores, minor EMI^+^ ions strongly confined in ion-sized micro-pores will undergo all kinds of the atomic-scale local microenvironments (local dielectric, inhomogeneity, topology, connectivity, distribution of the pore throats, metallicity, curvature and defects), and a resultant structure reorganization [5,6]. Particularly under the induction of the electric potential in pore sidewalls, the dispersion force between the molecules and sidewalls is left either commensurate with the coulombic interaction or even bigger enough to partly disrupt/de-coordinate the alternating coulomb array patterns of anions/cations, with both an appreciable co-ion paring generated in the first or nearest coordination shells around a central ion and image counter-charges arising in sidewall carbon atoms (Figure 4b). The local ion populations monotonically vary from the surfaces and exhibit both a maximum for cations and a minimum for anions at centers. Meantime, such in situ-induced image counter-charges at electrode surfaces can work as a robust compensator for charge of ions and thus as a strong driving force for the liquid ordering to further enable a newly 2D ordered planar structure of a co-ion chain of the cations/anions developing/aligning commutatively (viz., at the neighborhood of each other) in the 0.8-nm micro-pores, thereby self-amplifying the ion partitioning and surface charging. Such dielectric shielding effect results in the noted interfacial densification of the counter-ion conformations (the inter-molecular rearrangement) under polarization; and the local ratio of the quantities of ions of the different charges substantially deviates from unity although the aggregate volume occupied by ILs in electrode remains nearly invariable [38,45,46,47,48,49,50]. In this mono-tier confinement, the integral charges carried by a first adsorption tier can more precisely counterbalance those of the carbon pore sidewalls contributing to a far superior efficacy; affinity of ions in the first tier to the sidewall surfaces is not neutralized by the well-assembled abutting tier, thus causing these ions to reach the carbon surfaces more closely than outside the ultramicropores of C-0.25. Since the capacitance scales with the inverse of the distances between two charged planes [51], this contracted carbon atom-ion length (wetting length) along with the higher charge efficiency notably increases the capacitance to 54.7 F g^−1^ in the ion-sized pores of C-0.5, namely, just a 1.9 folds rise in specific surface areas can surprisingly bring about one order of the magnitude gain in capacitance. Vividly speaking, during charging the carbon atoms, the micropores transition from a fully irrelevant outlier in C-0.25 towards an electrochemically active participant for C-0.5. This microscopic origins also clarify that the image forces from the polarization of the electrode surface atoms by the ion charges in particularly confining pore structures can work as a pivotal integrant to exponentially screen out the coulombic repulsion energies between ions of the similar charges and so achieve the greater charges conserved in a suitably-sized microporous electrodes.

### 4.3. The Ion Partitioning on C-0.75

More surprisingly, a further modified KNO_3_ dosage can induce a certain amount of the 1.5-nm mesopores (about double the size of completely decoordinated EMI^+^ ions) while not compromising the ion-sized micropore (0.8 nm). In light of the aforementioned charge storage mechanisms of C-0.5, a 1.3-times gain of specific surface areas of C-0.75 relative to C-0.5 should have induced a capacitance of roughly 92 F g^−1^ rather than the dramatic increase to the anomalous experimental value of 158.9 F g^−1^. Put more strictly, a comparable capacitance between C-0.5 and C-0.75 should have been achieved because they both have the nearly same micro-pore surface areas and volumes (Table 1). This finding signally collides with a long-held belief concerning the ion sieving effect that only as the ion perfectly adjusts itself to pores to minimize free spaces available does the peak capacitance of ILs within the nanopores occur at a critical pore/ion size ratio of ≈1 [33,51]. In light of this view, the very atypical enhancement seen in C-0.5 should have been obscured for C-0.75 with the much wider PSD from 0.8 to 1.5 nm, in that the experimentally tested capacitance could be averaged over a wide pore diameter, in sharp contrast with its actually surprising increase. This explicitly unveils that the newly created 1.5-nm mesopores (basically responsible for the capacitance improvement) underlie a more efficient but distinct energy storage mode at play. In addition to the single ion tier confinement in ion-sized pores of 0.8 nm, the 1.5-nm pores ideally double the ion size and thus almost “seat” two ion tiers via a most efficient ion packing not just impacting the pore accessibility (Figure 4c). That is, the mono-/bi-tier confinements co-exist and robustly cooperate in C-0.75. In essence, the abnormal capacitance modification must stem from the highly reinforced DoC of the adsorbents, by which the detrimental overscreening of the long-range interionic electrostatic correlation networks is annihilated to yield a more amplified ion partitioning (featured by a larger capacity). By reinforcing the DoC, a stronger electrostatic counteraction (by the carbon pore sidewall) of coulombic repulsion interactions between ions of the same charge (namely the stronger net surface charging of carbon atoms) can proceed to allow for the “geometrically confined preferential” ion stacking in the carbon nanospaces. 

Next, the deeper reasons about how such introduced 1.5-nm nanopores synergically collaborate to improve the capacitance is tentatively explained from the forthcoming important respects.

### 4.4. Reasons for the Distince Ion Partitioning on C-0.75

#### 4.4.1. DoC of Ions Inside all Electrodes

**Figure 5:**
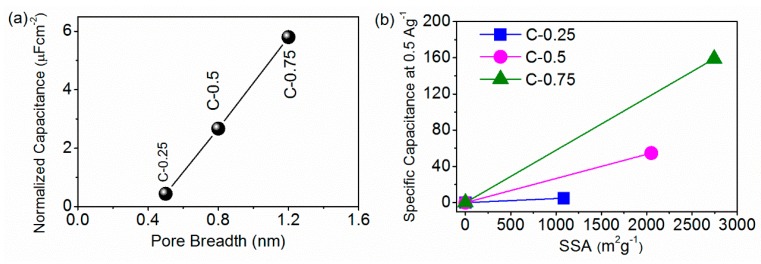
(**a**) Surface-normalized capacity versus pore size. (**b**) Specific capacity at 0.5 Ag^−1^ versus SSA (specific surface area), and slopes of the lines are proportional to relative permittivity ε (based on C = εS/d).

DoC from the local pore structure effects must closely correlate with the overall capacitance distribution per unit specific surface area (C_sur_) or per unit specific pore volume (C_vol_). Thus, C_sur_ and C_vol_ can be used to capture the underlying physics of actually involved electrochemical processes [8,52]. As seen in Table 3, at 0.5 A g^−1^, C_sur_ drastically rose from 0.44 µF cm^−2^ for C-0.25 and 2.67 µF cm^−2^ for C-0.5 to 5.8 µF cm^−2^ for C-0.75 while C_vol_ also remarkably increased from 11.9 F cm^−3^ for C-0.25 up to 66.5 F cm^−3^ of C-0.5, followed by 122.0 F cm^−3^ for C-0.75, hinting that more charges are stored in per unit volume or the surface of the ion-accessible nanopores. Even at a high current density of 10 A g^−^^1^, C_sur_ could be stabilized at 3.20 µF cm^−^^2^ for C-0.75, greater than 0.16 µF cm^−^^2^ for C-0.25 and 1.09 µF cm^−^^2^ while C_vol_ could be maintained at 65.9 F cm^−^^3^, in sharp contrast with 4.2 F cm^−^^3^ for C-0.25 and 27.2 F cm^−^^3^ for C-0.5. This gradual evolution of DoC unraveled an atypical capacitance scaling for the pore size from C-0.25 to C-0.75 (Figure 5a). In nature, both C_sur_ and C_vol_ are equivalent to the mean distribution of charge per carbon atom in the local pore structures. The overall quantities of charges triggered off on the electrodes were proportional to the absolute number density difference between counter-ions and co-ions and seldom scale with contents of electrolytes electrosorbed into nanopores. The notably rising C_sur_ and C_vol_ value means a significantly increased fraction of the carbon atoms (or bulky regions) accessible/exposed to counter-ions, that is, a plummeting even inappreciable co-ion quantity in proximity of carbon atoms (Figure 4c). Since the co-ions could trigger the counter charges over vicinal carbon atoms to reduce the charge population, a greatly up-regulated charge separation could occur to C-0.75. The above data undoubtedly disclosed that the newly-created 1.5-nm mesopores could apparently augment both the differential ion number densities of anions/cations and the mean DoC of adsorbed ions in nanopores, namely, the larger electroactive sites with a far greater DoC for more electrochemically participatory ions. Ions on centers having high DoC can be visually conceptualized as ions within much narrower carbon nanopores possessed of special local confining surroundings [8]. The largest DoC uncovered both a smallest equivalent pore size of C-0.75 (even much below the case for ion-size pores of C-0.5) and the highest pore volume utilization by ions under the extreme nanoconfinement. The local electrostatic potential became extremely uneven when the pore size puts on or below a par with the ion dimension. Due to the electronic polarizability of the metallic carbon pore walls of C-0.75, the resultantly strengthened electrostatic interplay between ions and sites equipped with a high DoC could impulse the counter-ions to head their way into and firmly occupy such highly confining locations, thereby accelerating the ion swapping with bulk ions and thus augmenting both local ion densities and pore occupancy (Figure 4c). The resulting EDL microstructures at such high DoC were dominated by the counter-ion distribution. Any preferential adsorption of cations or anions could break down the charge neutrality, hence inducing the net charges at surfaces. Thus, the corresponding net electrode surface image charges (triggered by coordination with these highly restricted ions) were more localized or trapped at the centers with the big DoC, while the coulombic electrostatic interactions got shielded to the greatest extent. Since these sites with high DoC could produce local dielectric inhomogeneity near interfaces, a self-energy and ion-image charge effect emerged, in turn notably stepping up the interface polarization [53,54]. Such perturbed equilibrium between anions and cations in combination with the increased counter-ion levels ensured a lowest energy dissipation caused by the counter-ion repulsion, thus maximally storing the local electrode surface charges and most effectively shielding the electric fields of the highly confined ions. 

Pursuant to basic principles of SCs: C = εS/d, wherein S is the electrode surface areas accessible to electrolyte ions, ε designates relative dielectric permittivity (normally, at the same test conditions, the ε only relates to the intrinsic properties of electrode materials and drops with the increased template dosage in our system) and d means the thickness of the EDLs (a value of a few angstroms, merely relating to the electrolytes ions and solvent dimensions) [51], as indicated in Figure 5b, the slopes of lines (ε/d) were proportional inversely to d and thus d did not keep stable but was made lower with the increasing template dosage, namely, d_C-0.25_ > d_C-0.5_ > d_C-0.75_. This highly shortened wetting length (d) could explain that the ions (mainly counter-ions) under the stronger nanoconfinement (namely, a much smaller equivalent pore width than the case for the ion-size pore of C-0.5) must reside much nearer to carbon pore sidewalls than their solvated semi-diameter, facilitating formation of a dense ion layer. So, a regulated amplitude of a desired DoC enhancement must fall through unless, in any case, partial or total neighboring coulombs of a single ion in its first/second coordination shell (the basic coulomb ordering structure) require mal-forming, twisting, liberating or stripping off while the coordination envelop is squeezed through or inserted at centers with high DoC in a much analogous manner that a balloon misshapes when entered across an opening below its equilibrium dimension. In this case, the Bjerrum length between two adjacent ions obviously falls, unveiling that the DoC stays highly impressionable to the extent of the reorganization and decoordination of the in-nanopore ions from weak to strong nanoconfinement. The more nanoconfined, the more undercoordinated/rearranged. Since the surface image charges could proportionally balance those of the counter-ions abutting them, the more drastically even completely decoordinated ions (viz., with the smaller coordination number of the co-ions around every counter-ion or the unpaired counter-ions) were able to more polarize the electrode atoms to induce the maximum charges than the more highly coordinated ions. In effect, a thorough decoordination (coordination number = 0) or single-coordination (coordination number = 1) most probably happens exclusively if the ion dimension rigidly approaches the pore width; and such nanopores appeared more likely in C-0.5 and in the 0.8-nm pores of C-0.75 than in 1.5-nm pores of the two-ion size in C-0.75, finally inducing the highly averaged coordination shell partners over wide PSD. Seeing that increment in the pore volumes and surface areas from C-0.5 to C-0.75 was not quite coextensive with their C_sur_ and C_vol_ rise, C-0.75 seemed incompetent to attain such high charge density simply via electro-sorbing the high amounts of the relatively low charge inducers (somewhat highly coordinated ions) in an identical ion packaging pattern with C-0.5. Thus, a high DoC for C-0.75 must be caused by forming a superionic state high-density ion clustering within the 1.5-nm pores wherein ions of similar charges approached/interacted closer than in 0.8-nm pores, with a more repulsive ion-ion electrostatic energies (Figure 4c). The rich structural anisotropy of anions and cations (such as intra-molecular freedom degree and concealed charged groups) allowed the quasi-crystalline ionic cluster structures to occur (especially under external field and confinement) by structural rearrangement and phase change (Figure 4c), as analogized to nanoscaled-freezing [55], -crystallization [56], -melting [57] and so forth. These clustering structures with regard to one single unpaired anion can become more helpful in interacting with the pore walls and inducing the higher image forces within local electrode atoms to separate/store the charges. Accordingly, pivotal roles that these newfound small mesopores play consist in intensifying not only a total charge offsetting mechanism through quickening an in-nano-pore ↔ ex-nanopore ion exchange (an electro-kinetic pumping characteristic) but also a local phase conformation rearrangement/transformation with the great propensity for the ions charged oppositely to the electrode surfaces (counter-ions) to occupy the strongly nanoconfined situations. 

#### 4.4.2. The EDL Overlapping Behavior

The promoting impact from the 1.5-nm mesopores could also be illustrated on grounds of the well-established EDL overlapping theory [58,59,60,61,62,63]. As stated above, the EDLs of ILs adjoining the open planar electrode surfaces are distinguished by multiple alternating stratifications of counter-ions and co-ions outstretching (no more than a few nanometers thick) far away from electrified carbon surfaces; and their concomitant parameters such as the local permittivity, the charge/ion number distribution and potentials can likewise feature an analogous alternating behavior. Accordingly, this decreasingly oscillatory layering of the positive and negative charges can be deconvoluted into a series of the EDL capacitors where the corresponding capacitance can be dictated by *1/C* = ∑*1/C_m_*, where *C_m_* means the capacitance of the layer m [54]. This unique signature resembles the wave’s peculiarity in physics, so EDLs over open surfaces of C-0.25 can be deemed as akin to a sterically damping wave. This sterically fading “wave” can get delineated vividly via its number population distribution of ionic species from ILs around the open planar electrode surfaces under the employed voltages. When two EDLs develop and evolve at two charged and parallel walls, the inevitable overlapping occurs constructively or destructively. The resultant EDL microstructures from each wall must interface and evolve mutually in a way complying with the wave’s interference laws that two standing waves superpose with each other into a consequent one, accompanied by the districts of the positive (crests synchronizing with crests) and negative (crests contemporizing with troughs) interferences. As a matter of fact, geometry of the 1.5-nm pore can be circumstantially modeled as the narrow 1.5-nm-wide slit. Its wholistic EDLs at the tangential applied potentials can ideationally originate from the vector superposition of two independent EDLs near two symmetrical surfaces opposite to one another. Given the fact both that the typical thickness of every unit layer of alternated counter-ion and co-ion layers approximates ion diameter (steric length) of 0.76 nm and that anions and cations tend to cumulatively populate the central plane of the 1.5-nm-slit-like pore to minimize the free energy by the maximal solvation, an individual EDL in this notional slit must be symmetrized at central positions and truncated at each side. A corresponding spatial ion/charge apportionment interrelates with the superimposition of the ion apportionment at both the opposing open planes, balanced at the slit heart. Thus, there must emerge the most remarkable “resonance” of the ion population for the identically charged ions, as described by the interference of the wavevector dissemination. That is, double ion-sized pores assure that the overlapping EDLs from two opposing sidewalls encounter a maximally positive interference in terms of density of one ion from one pore sidewall so as to maximally superpose and resonate with that of the identical-charged ion from another sidewall. Such positively maximized interference must represent a most reinforced ion packaging/distribution. The maximal ion density distribution shapes the local dielectric constant, so the optimal interface polarization and capacitance. More significantly, this maximal EDL overlapping can intensify various electrokinetic phenomena (taking place within the porous media under an electric field), which permit the enhanced mass diffusion and transport. Normally, these electrokinetic phenomena include the electrokinetic surface conduction, the electro-osmosis, electrophoresis, the streaming current and so forth. These electrokinetic effects can, further, prominently facilitate an ion swapping and self-alignment effect during the fast charging/discharging to enhance energy storage. 

#### 4.4.3. The Spatial Connectivity Influence

The notable impact of the 1.5-nm mesopores on expediting the charging kinetics can be further understood based on the spatial connectivity of electrode materials. On the one hand, an appreciable development of small-sized mesopores can trigger the contraction of domains of micropores and the creation of the plentiful hetero-junctions between the 1.5-nm mesopores and the ion-sized but much shorter/less tortuous 0.8-nm micropores (than these of C-0.5), notably shortening the incoming ion’s transmission trajectories from exteriors to interior sites with high DoC. Such big pores can act as the “expressways” concatenating the smaller pores, offering myriad pore entrances with the edge effects and altering the nearby electrical potentials. By means of heterojunctions, the mesopores interconnect and interpenetrate with the micropores to generate a pervious/continuous network. Thus, the spatial anisotropy of the ion diffusions can be facilitated. For example, the outgoing/incoming ions could find more ways out of/into these “single/double ion-size” carbon nanopores to address the trade-off between the overfilling and defilling phenomena that substantially decelerate the charging dynamics [64]. 

On the other hand, the mesopore width decides that ILs in mesopores are more liable to adopt a bi-tier permutation fashion, namely the bi-tier confinement. Ions must be more densely/compactly partitioned inside the mesopores with either the partial stripping off or the superposition of their coulombic ion coordination envelope. Within the bulk ILs, the ions develop into a 2D matrix with the co-/counter-ions interlinked mutually like an ionic crystal. Motion of the ions in this circumstance necessitates a sizable activation energy for extricating the co-/counter-ion couples or breaking their chemical binding, so the ion transportation must stay sluggish. As long as the numerous counter-ion influxes emerge in the sites with the high DoC, the ideal interlocked co-/counter-ion matrix will vanish immediately, and ions can travel more liberally and freely. If the suitably sized mesopores are devoid, this quickened in-nanopore self-diffusion will be seriously attenuated because the counter-ions out of ion-sized micropores are directly correlated to or faced up with several tiers of highly short-range coulombic ordering of bulk ILs. As a result, ions have to plough their way through a sea of oppositely charged ions to overwhelm the coulombic interactions, and the ion migration is made slower. When a buffering reservoir exists, these outgoing ions develop into a dual-tier in 1.5-nm pores. In this case, such interionic electrostatic tethering is maximally disturbed by the ion-wall interactions and even vanishes due to high DoC, while a good deal of the pertinent ions and the relevant coordination or conformation variations are ensured. The extremely de-coordinated counter-ions from strong mono-tier confinement turn into a metastable near-Wigner crystal with a tiny number of the co-ions as the impurities under the comparatively weaker dual-tier confinement [64]. That is, both the long-range electrostatic screening and the new short-range self-layering/patterning of the 2D ordered planar structures could simultaneously come true in a highly combined mono-/bi-tier nanoconfinement. Meanwhile, this process is accompanied by the formation of the quasi-liquid crystals, a metastable intermediate phase status between the fully periodic and completely disordered media. For example, the ethyl group in the cations of EMIMBF_4_ can turn into a butyl group while anions can readily switch between BF_4_^−^ and TFSI^−^. This unique crossover structural trait could not only remove the long-short transportation through bulk electrolytes as arises in C-0.5 but also effectively shuttle the ions between two kinds of pores. In theory, this confinement-triggered phase transition is desired if the surface energies can stabilize the disfavored phase via offsetting the bulk free energies. This brings about a shift of the phase transition, as discussed here. Actually, ILs around nanointerfaces with the highly metallic carbon sidewalls can be deemed as a semi-infinite ionic crystal. The web of the image charges constructs a crystal configuration possessed of an almost ideal symmetry relative to the realistic half-lattice. Thus, electrostatic contribution to surface free energy gets removed, with the overall interface system conducting itself like a single bulk lattice. Definitely, such a scenario must utterly call for an ideally symmetric crystalline conformation. Such revocation stands slim chances of emerging under the low DoC. This is to say, the newfound semi-infinite (quasi-) crystal of the predominant counter-ions does have recourse to a considerably smaller surface energy around the interphase boundaries with the conducting carbon sidewalls of C-0.75. This manifests that a “micro-environmentally nano-confined” preferable ion reconfiguring of a quasi-crystalline nature is thermo-dynamically preferred. In summary, this homeostatic molecular conformation transition amidst the various ion coordination structures under the bi-/mono-tier confinements can reversibly switch with the considerably smaller energy barriers during the charging/discharging, thus contributing much to a distinct charge storage from both the conventional EDL compression and the battery-involved processes. 

#### 4.4.4. The Supports from the CV Data

Lastly, such molecular structural changes in dual/single-tier confinements can be supported well by the detected high-voltage CV peaks (Figure 3a). Accurate source of these clear-cut bands from CV plots still remains elusive but can be reasonably excluded neither from the pseudo-capacitance effect and nor from a substance dissociation (almost equicrural CP curves). These peaks must reference the reversible structural perturbation under a high DoC, accompanied by the forfeiture/recuperation of energies during the charging/discharging. For one thing, a high electrochemical working window of 4 V and specific capacitance as high as 158.9 F g^−1^ can translate into a global conserved charge density over 635.6 Cg^−1^, and the aggregate quantity of the specific electricity conserved within the current devices outstrips the homologues of the commercial EDL capacitors by over one magnitude. These values seem too great for the routine compact EDLs consisting of ILs with the giant ion dimension. Besides, the nominal area occupied by the per molecule around electrochemical interphases reaches 0.68 nm^2^, even smaller than the molecular size. This means that the per carbon electrode surface area (nm^2^) can accommodate as high as 1.6 ions of ILs (an equivalent amount of image charges over carbon phases), which reveals a close-to-dense cation tier on carbon pore surfaces. It seems rather unfeasible for the huge-sized ILs ions to develop such a sizeable EDL compression if no foreign organics or water molecules exist. Different from traditional solvent-based systems, the wholistic cation/anion population must keep identical with its bulk level, so the electricity must fall short of being conserved inside such a highly condensed ionic tier only via eliminating/extracting the electro-neutral molecules. Instead, the conformational transformations (mainly towards stripping off the ion coordination shells, distorting the coulombic ordering, delocalizing the intramolecular/interatomic electrostatic charges and coulombic larger voltage locations in CV plots) can turn into the most presumable scenario. This structural phase variation can advance over several tiers and be not over-screened by the interionic correlations at the strong confinements. Accordingly, such a phase transition mode can respond ultra-sensitively to the pore size/volume instead of the specific surface areas, as clearly verified both by the fact that two thirds of overall charges in C-0.75 are conserved within 1.5-nm nanopores whereas one third in the ion-sized micropores (0.8-nm pores) and by the fact that the non-linear dependence of capacitance on the specific surface areas was clearly observed in a homogenous series of nanocarbons with the perfectly-controlled narrow PSD from C-0.25 to C-0.75 (Figure 2a). For another thing, on an enthalpy scale, the cell voltage range wherein the peaks take place extensionally corresponds to the considerably larger transformation enthalpy varying from 200 to 400 kjmol^−1^. Although this enthalpy level notably surpasses the values of covalent bonds, hydrogen bonds, coulomb isolation, inter-molecular interaction and van der Waals forces, it still belongs to the scope of the salt melting, other collective properties, the drastic configuration variations with their ion coordination shell and so forth (Figure 3a). It again corroborates that the CV peaks are certain to reference some certain high-energy structural transformations. In essence, these events closely correlate with a local in-nanopore interionic/intraionic arrangement in the Angstrom level rather than the low and diffusion/viscosity-limited intercalation, pseudocapacitance battery or conversion-type behaviors. Thus, it performs a quasi-capacitive rate response and the enhanced power handling capability. The above structural transformations can strictly agree well with latest-revealed anomalous phenomena with a complete confinement-induced phase transition of ILs within nanospaces, e.g., nanoscaled thawing, nanoscaled superlubricity, nanoscaled recrystallization, nanoscaled electro-wetting, nanoscaled capillary freezing, nanoscaled friction, nanoscaled capillary condensation and so forth [55,56,57].

## 5. Conclusions

Filling ionic liquids within the porous carbon electrodes maintains both efficient conservation of the high electricity in supercapacitor and the rapid velocities with which the charging/discharging proceeds to accomplish the excellent power behavior. However, rare works have clarified material attributes, which dominate the energy storage efficacy and charging dynamics. Herein, a fresh type of salt-templating nanocarbons with hierarchically varied texture properties were facilely derived from the renewable biomass and assessed as a probe electrode to tentatively delineate the electrochemical events of SCs employing high-voltage ionic liquid (EMIBF_4_) as an energy carrier fluid. The significant outcomes clearly elucidated that the generation/condensation of conventional EDLs could only in a small part depict the charging behavior of the nanoporous electrode configurations, while a peculiar high-voltage structural transformation in the aptly sized nanopores (a mixture of the micropores and small mesopores) can substantially contribute to global energy contents. This novel mode was accompanied by both the partial stripping of ion coordination shells and coulombic conformational rearrangement made probable by the strong image forces from polarization of the electrode surfaces. Its remarkable efficacy over those of planar graphite electrodes and of the pure micropore inclusion of single ion was attributed to the high confinement degree. The high confinement degree could evade the harmful interference/overscreening effects and intensify the reversible structural oscillations. Interestingly, this strong nanoconfinement could be readily conferred just via modulating a hierarchical local pore microstructure, for instance, the perfectly controlled narrow pore size distribution, which mainly combined the ion-sized micropores (0.8 nm) with double ion-sized mesopores (1.5 nm). Manipulating the charging mechanisms ultimately enabled tailoring the specific energy/power property of our optimally designed SC cells (88.3 Wh kg^−1^ at 1 kW kg^−1^ and 47.7 Wh kg^−1^ at 20 kW kg^−1^) into batteries’ ranges. Besides, a satisfactory capacitance retention of 83.3% even over a 5000 cycles at a rather high current density of 10 A g^−1^ and an exceptional rate retention of 54.0% from 0.5 to 10 A g^−1^ could be achieved. This finding of a non-classic and powerful electrochemical process built upon high-energy, reversible and quick phase reconfigurations offers the great opportunities toward tackling Ragone conflicts and augmenting the supercapacitive properties simply through further optimizing the nanoconfinement-effect-induced phase structure oscillations of the compositionally tuned ILs within the precisely modulated carbon nanopores from the micro- towards mesoscales.

## Figures and Tables

**Table 1:** The textural and compositional properties of samples.

Samples	S_total_ (m^2^g^−1^) ^a^	S_meso_ (cm^2^ g^−1^)	S_micro_ (cm^2^ g^−1^) ^b^	V_total_ (cm^3^ g^−1^) ^c^	V_meso_ (cm^3^ g^−1^)	V_micro_ (cm^3^ g^−1^) ^b^	Nitrogen Contents (at. %) ^d^
C-0.25	1084.3	45.5	1038.8	0.403	0.001	0.402	9.45
C-0.5	2050.3	280.2	1770.1	0.825	0.107	0.718	5.52
C-0.75	2744.6	880.5	1864.1	1.304	0.478	0.826	1.68

^a^ calculated via the multi-point BET (Brunauer-Emmett-Teller) method; ^b^ gauged by the t-plot way (adsorption thickness was controlled at 0.3–0.5 nm); ^c^ computed from the QSDFT (quenched solid density functional theory) equilibrium model and ^d^ identified through XPS measurement.

**Table 2:** A summary of the key performance parameters such as specific capacitance, rate capacity, energy-power density and capacity retention.

Samples	Specific Capacitance (F g^−1^) at 0.5 A g^−1^	Specific Capacitance (F g^−1^) at 10 A g^−1^	Rate Capacity from 0.5 to 10 A g^−1^	Energy Density (Wh kg^−1^) at 1 kW kg^−1^	Energy Density (Wh kg^−1^) at 20 kW kg^−1^	Capacity Retention at 10 A g^−1^ over 5000 Cycles
C-0.25	4.8	1.7	35.0%	2.7	0.94	100%
C-0.5	54.7	22.4	41.0%	30.4	12.4	87.0%
C-0.75	158.9	85.9	54.0%	88.3	47.7	83.3%

**Table 3:** The wholistic capacitance distribution per unit specific surface area (C_sur_) or per unit specific pore volume (C_vol_).

Samples	C_sur_ at 0.5 A g^−1^	C_sur_ at 10 A g^−1^	C_vol_ at 0.5 A g^−1^	C_vol_ at 10 A g^−1^
C-0.25	0.44 µF cm^−2^	0.16 µF cm^−2^	11.9 F cm^−3^	4.2 F cm^−3^
C-0.5	2.67 µF cm^−2^	1.09 µF cm^−2^	66.5 F cm^−3^	27.2 F cm^−3^
C-0.75	5.80 µF cm^−2^	3.20 µF cm^−2^	122.0 F cm^−3^	65.9 F cm^−3^

## Supplementary Materials

The following are available online at https://www.mdpi.com/2079-4991/9/12/1664/s1. Figure S1: N_2_ adsorption–desorption isotherm curves; Figure S2: Full-scale XPS spectra of C-0.25, C-0.5, and C-0.75; Figure S3: CV curves at 200 mVs^−1^; Figure S4: The IR_drop_ value at various current densities; Figure S5: Stability test conducted at 10 A g^−1^ for 5000 cycles in EMIBF_4_ ILs at 4 V; Table S1: Comparison of the cyclic life of C-0.75 with those of some advanced carbon/ion liquid systems reported previously.
