# Supplementary material for: Studies on Possible Ion-Confinement in Nanopore for Enhanced Supercapacitor Performance in 4V EMIBF4 Ionic Liquids
Source: Nanomaterials (Basel). 2019 Nov 22;9(12):1664. doi: 10.3390/nano9121664 (PMC6956350; doi:10.3390/nano9121664)
Supplement: Supplementary file 1 [file nanomaterials-09-01664-s001.pdf]

Supplementary Materials: Studies on Possible Ion-Confinement in Nanopore for Enhanced Supercapacitor Performance in 4V $EMIBF_4$ Ionic Liquids

Materials preparation

Four steps, including sol-gel, freeze-drying, annealing, and water washing, were involved in the synthesis of porous biocarbon. Typically, C-0.75 was synthesized as follows. Firstly, 1 g of gelatin and 0.75 g of KNO_3 were dissolved in 20 mL of hot water at 80 °C to form a transparent sol. After cooling to the room temperature, the sol was placed in a refrigerator at 4 °C for 12 h and then transferred to −20 °C for additional 12 h. Then, the frozen jell was placed in liquid nitrogen for about 30 s, followed by freeze-drying in a lyophilizer at −80 °C, 1 Pa, for 36 h to get a light aerogel. After that, the annealing process was conducted at 800 °C for 1 h in pure Ar gas with a flow rate of 300 mL/min and a heating rate of 5 °C/min. The final product was obtained by direct water washing and drying at 60 °C for 24 h. Samples synthesized with different dosages of KNO_3 salt (0.25, 0.5, 0.75 g) calcined at 800 °C were also obtained via the above process and marked as C-0.25, C-0.5, and C-0.75, respectively. Traces of NO_x emission during carbonization could be easily removed via a direct water adsorption method. All reagents are used as the analytical reagents and purchased from Guoyao Chemical Co., Ltd (Shanghai, China). All the chemicals were used as received without further treatment.

Electrochemical evaluation

The electrode slurry was prepared by mixing 80 wt % active materials, 10 wt % acetylene black, and 10 wt % PVDF binder in NMP solvent. Then, the slurry was loaded on the round-disk Ni foam (1 mm in thickness, 1.1 cm in diameter) with a mass loading of about 3 mg cm^2. After vacuum-drying and compression under 10 MPa for 30 s, the working electrodes were prepared.

Test in $EMIBF_4$ electrolyte: A symmetric two-electrode coin cell was assembled in pure Ar glove boxes with concentrations of both oxygen and moisture lower than 0.1 ppm. A Whatman membrane (680 μm in thickness), made from glass microfiber (type: GF/D1823-047), was placed between two electrode sheets with the same loading, and all of them were compressed together and sealed in a 2025-type coin cell. Electrochemical performances including CV, GCD, and EIS were evaluated. The voltage range was 0–4 V in the CV test with different scan rates ranging from 20 to 200 mVs^{-1}. GCD tests under various current densities of 0.5–10 Ag^{-1} were also performed between 0 and 4 V. The electrochemical impedance spectroscopy measurement was carried out with an electrochemical analyzer within a frequency range of 10^5–0.01 Hz.

The specific capacitance ($C_{electrode}$, Fg^{-1}) based on each electrode was calculated by the formula:

$$C_{electrode} = 4I\Delta t/mV, \quad (1)$$

where I, Δt, m, and V are the constant current (mA), discharge time (s), total mass of both carbon electrodes (mg), and voltage window (V).

The specific energy density (E, $Whkg^{-1}$) was calculated on the basis of the equation:

$$E = C_{cell}V^2/7.2 = C_{electrode}V^2/28.8 \quad (2)$$

The specific power density (P, Wkg^{-1}) was obtained according to the formula:

$$P = E/\Delta t \quad (3)$$

Material characterization

The morphology and structure of the samples were characterized by a scanning electron microscope (SEM, JSM 7401F, JEOL Ltd., Tokyo, Japan) operated at 3.0 kV and a transmission electron

microscope (TEM, JEM 2010, JEOL Ltd., Tokyo, Japan) operated at 120.0 kV. X-ray photoelectron spectroscopy (XPS) measurements were carried out on Escalab 250xi (Waltham, MA, USA). All XPS spectra were calibrated at 284.8 eV using C 1s line, and the raw data were fitted by XPSPEAK program (XPS peak 4.1, Taiwan). The N_2 adsorption/desorption isotherm was recorded by an Autosorb-IQ2-MP-C system (Boynton Beach, FL, USA). The specific surface area (SSA) was obtained using the multipoint Brunauer–Emmett–Teller (BET) method, and the pore-size distribution (PSD) was obtained via the Quenched Solid Density Function Theory (QSDFT) and the equilibrium model.

Supplementary data

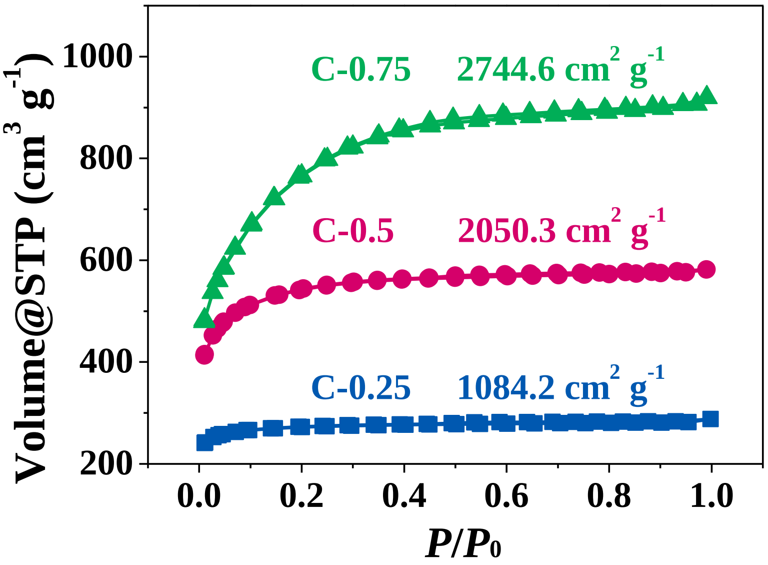

Figure S1. N_2 adsorption–desorption isotherm curves.

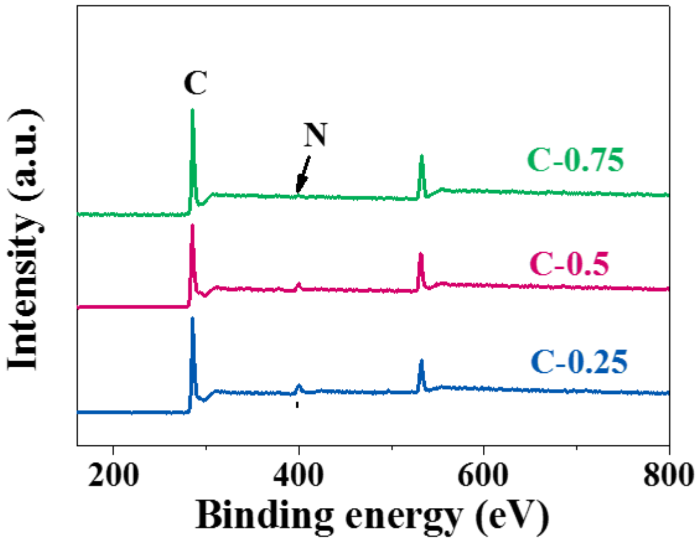

Figure S2. Full-scale XPS spectra of C-0.25, C-0.5, and C-0.75.

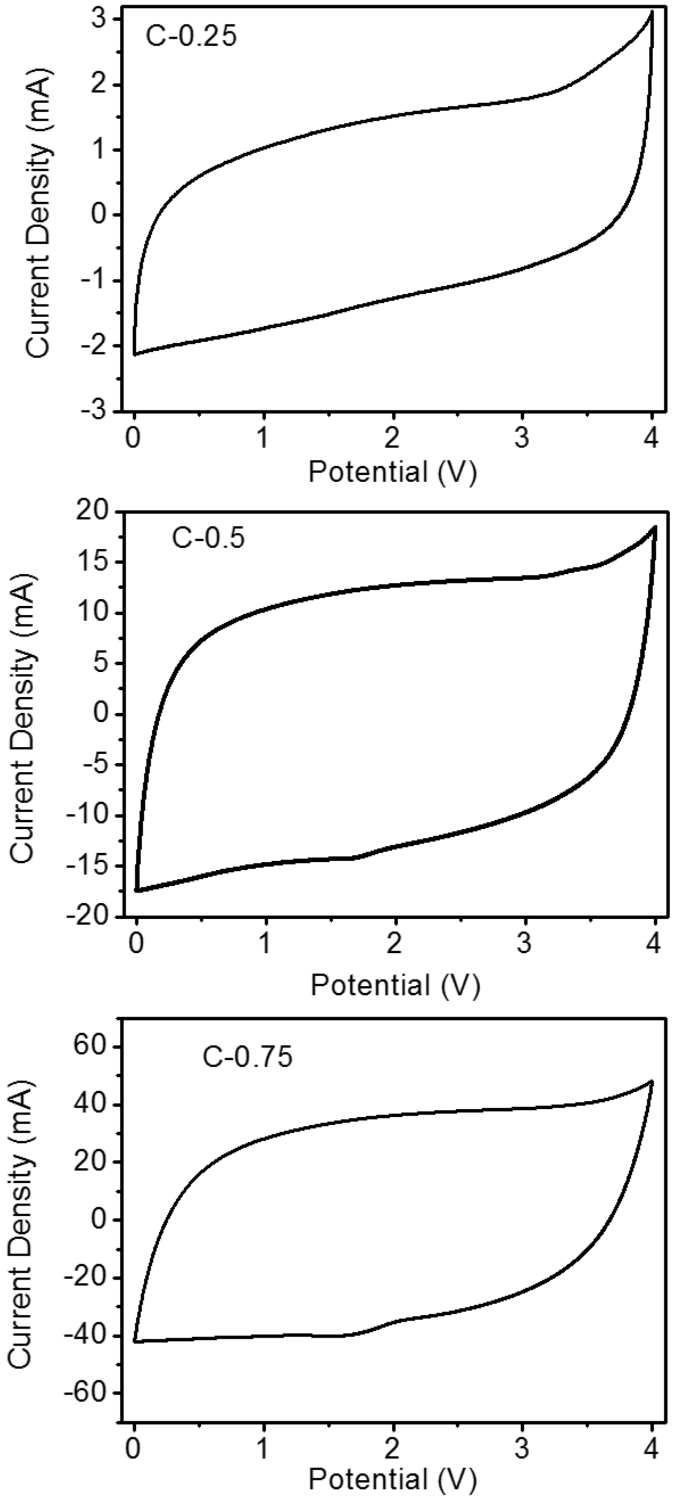

Figure S3. CV curves at 200 mVs^{-1}.

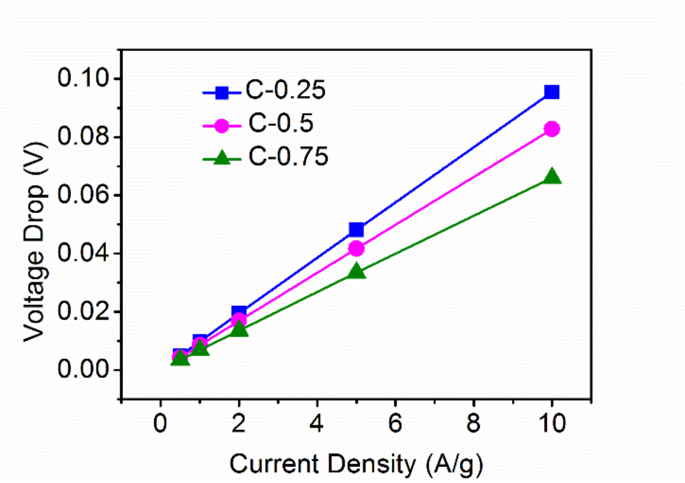

Figure S4. The IR_{drop} value at various current densities.

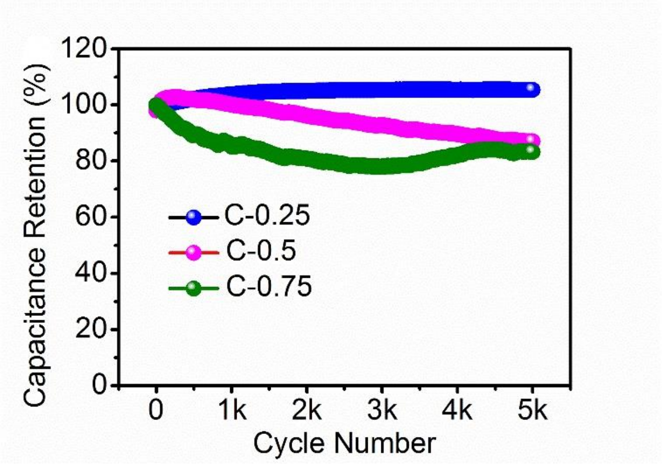

Figure S5. Stability test conducted at 10 A g^{-1} for 5000 cycles in $EMIBF_4$ ILs at 4 V.

To demonstrate the cyclic lifespan, a 5000-time continuous charge–discharge process was carried out at a very high current density of 10 A g^{-1}, as seen in Figure S5. Finally, a satisfactory capacitance retention of 83.3% (only 0.33% decay per cycle) was attained for C-0.75, in sharp contrast with the counterparts of constant 100% for C-0.25 and 87.0% for C-0.5. This stability evolution can be understood considering the structural motifs of the samples. In C-0.75 compared to C-0.25, both porosity and hierarchy highly increased, with considerably higher mesoporosity contribution (Table 1 within the manuscript). To be specific, C-0.25 was purely microporous, while C-0.75 was characterized by a mixture of mesopores and micropores. Hence, under a rather demanding cyclic condition of 10 A g^{-1}, destruction of the electrode material could occur, thereby causing capacitance degeneration. The opposite trend between lifespan and porosity in essence reveals a great challenge to simultaneously accomplish exceptional capacitance and stability by modulating the porosity. However, the porosity (mainly the pore size distribution, as fully explained in the main manuscript) imposes the main impact on capacitance improvement. Worse still, structural collapse will substantially exacerbate in the presence of heteroatom dopants. This is because the decomposition of unstable heteroatoms can deteriorate the structural fluctuation during repeated redox reactions. C-0.75 did contain a small quantity of N atoms (1.68%), although the pseudocapacitance of the N surface functional groups does not absolutely govern the capacitance contribution (as analyzed in the manuscript). The unstable N-5- and N-6-type bonding will undergo irreversible conversion and therefore fuel textural variation. Such signature often sparks off a dilemma for carbon-based materials in ion liquid-wetted supercapacitors, as has been also widely showcased in the literature.

Noteworthy, in spite of the poorer cyclability of C-0.75 relative to the other two samples, its capacitance retention was still acceptable. As a matter of fact, capacitance fading of highly electroactive porous nanocarbons has been ubiquitously observed in the literature, [1–17], and it was found that over 5000–10,000 cycles, their capacitance retention is commonly stabilized within a range of 80%–90 %, very similar to the case in our study. A thorough comparison between our data and the hitherto published lifetimes of some cutting-edge carbon/ionic liquid systems was made and is summarized in Table S1. Factoring in the important repertoire regarding synthetic tractability, sustainability, and inexpensiveness, C-0.75 could split the difference between capacitance and durability, leading to a commendable supercapacitive performance for ion liquid-filled porous carbons (Table S1). From Table S1, it can also be clearly seen that hetero-atom-doped nanoporous carbons usually present a relatively lower capacitance retention than non-doped porous carbons, evidencing that the dopant or surface functionalities do commonly play a detrimental role in cyclic stability. On the other hand, our results also indicate that manipulating both the morphologic tenability and the purity of exquisitely tailor-made porous carbon nanomaterials is crucial so as to ensure a prolonged cyclic life, while maintaining reinforced specific capacitance, particularly when operating in high-voltage electrolytes. Our further work will concentrate on how to enhance the textural robustness/tolerance of our optimized materials (C-0.75).

Table S1. Comparison of the cyclic life of C-0.75 with those of some advanced carbon/ion liquid systems reported previously.

Optimal Samples	Synthesis Method	Cycle Number	Electrolyte	Capacity Retention	Specific Capacity ($F\ g^{-1}$)	Refs
C-0.75	KNO_3-templated biomass (water washing)	5000 (10A/g)	$EMIBF_4$ (4V)	83.3%	158.9 ($0.5Ag^{-1}$)	Our work
mesoporous graphene nanoflakes	hexane pyrolysis over MgO	5000 (2A/g)	$N^+Et_4TFS^-$ (3V)	88.2%	105 ($0.5Ag^{-1}$)	Ref 1
modified graphene	modified Hummers' method	2000 (10A/g)	$EMIBF_4$ (4V)	92%	135 ($0.5Ag^{-1}$)	Ref 2
interconnected porous carbon nanosheets	KOH hydrothermal/carbonization process of reed	10000 (10A/g)	$EMIMBF_4$ (3V)	90%	147 ($1Ag^{-1}$)	Ref 3
nitrogen/sulfur co-doped reduced graphene oxide aerogels	modified Hummers' method	5000 (5A/g)	$EMIMBF_4$ (3.5 V)	80%	180.5 ($1Ag^{-1}$)	Ref 4
porous carbon nanosheets	NaOH/KOH coactivation	5000 (10A/g)	$EMIMBF_4$ (3.5V)	93%	169 (1A/g)	Ref 5
hierarchically porous sheet-like nanocarbons	modified Hummers method	5000 (5A/g)	$EMIMBF_4$ (3.5V)	88%	158.1 (0.2A/g)	Ref 6
carbon nanomesh constructed by interconnected carbon nanocages	carbonization of $Ni(NO_3)_2$ and sucrose (HCl washing)	10000 (5A/g)	$EMIMBF_4$ (3.5V)	90.6%	194 (1A/g)	Ref 7
N/S co-doped hierarchically porous graphene aerogel	modified Hummers method	3000 (2A/g)	$EEIMBF_4$ (4V)	77.2%	169.4 (1A/g)	Ref 8
Porous carbons	H_3PO_4-activation	5000 (5A/g)	$BMIMPF_6$	86%	162 (0.5A/g)	Ref 9
chestnut shell-based porous carbon	Zn-assisted activation (acid washing)	2000 (1A/g)	$BMImBF_4$ (2V)	71.5%	134.6 (0.1A/g)	Ref 10
N, O codoped carbon nanosphere	diaminobenzidine as precursor and KOH activation	10000 (1A/g)	$EMIMBF_4$ (3V)	92.8%	45.3 (1A/g)	Ref 11
nitrogen-doped hierarchically porous carbide-derived carbon	polymer derived silicon carbonitride route	1600 (1A/g)	$EMIMBF_4$ (3V)	80%	129 (0.1A/g)	Ref 12
graphene and porous nanocarbon composites	thermolysis of PVDF, GO, and KOH.	5000 (5A/g)	$EMIMBF_4$ (3V)	85%	185 (0.5A/g)	Ref 13
Functional porous carbon nanospheres	NaCl/$ZnCl_2$ salt templating of urea and tannic acid	10000 (20 $mAcm^{-2}$)	$PYR_{14}FSI$ (3.5V)	90%	110 ($2mAcm^{-2}$)	Ref 14
activated graphene	modified Brodie method (KOH activation)	5000 (100 mV^{-1})	$EMIMBF_4$ (3.5V)	75.9%	131.5 (1 mV^{-1})	Ref 15
carbon nanospheres hanging on carbon nanotubes	chemical vapor deposition method	10000 (10A/g)	$EMIMBF_4$	92.7% (3V)	121.5 (0.2A/g)	Ref 16
graphene–CMK-5 composite	modified Hummers method + SBA-15 templating (HF treatment)	2000 (2A/g)	$EMIMBF_4$ (3.5v)	90%	144.4 (0.2A/g)	Ref 17

References

1. Ekaterina, A.; Arkhipova, Anton, S.; Ivanov, Konstantin, I.; Maslakov, Alexander, V.; Egorov, Serguei, V.; Savilova, Valery, V. Lunin. Mesoporous graphene nanoflakes for high performance supercapacitors with ionic liquid electrolyte. *Microporous and Mesoporous Materials*. **2019**, doi:10.1016/j.micromeso.2019.109851.
2. Qingguo Shao, Jie Tang, Yuexian Lin, Jing Li, Faxiang Qin, Kun Zhang, Jinshi Yuan, Lu-Chang Qin. Ionic liquid modified graphene for supercapacitors with high rate capability. *Electrochim. Acta*, **2015**, *176*, 1441–1446.
3. Dong Zhou, Huanlei Wang, Nan Mao, Yanran Chen, Ying Zhou, Taiping Yin, Hui Xie, Wei Liu, Shougang Chen, Xin Wang. High energy supercapacitors based on interconnected porous carbon nanosheets with ionic liquid electrolyte. *Microporous and Mesoporous Materials*, **2017**, *241*, 202–209.
4. Yujuan Chen, Li Sun, Zhaoen Liu, Yuyang Jiang, Kelei Zhuo. Synthesis of nitrogen/sulfur co-doped reduced graphene oxide aerogels for high-performance supercapacitors with ionic liquid electrolyte. *Mater. Chemistry and Physics*, **2019**, *238*, 121932–121941.
5. Dewei Wang, Lang Xu, Jiawang Nai, Tao Sun. A versatile Co-Activation strategy towards porous carbon nanosheets for high performance ionic liquid based supercapacitor applications. *J. of Alloys and Compounds*, **2019**, *786*, 109–117.
6. Hai Su, Haitao Zhang, Fangyan Liu, Fengjun Chun, Binbin Zhang, Xiang Chu, Haichao Huang, Weili Deng, Bingni Gu, Hepeng Zhang, Xiaotong Zheng, Minhao Zhu, Weiqing Yang. High power supercapacitors based on hierarchically porous sheet-like nanocarbons with ionic liquid electrolytes. *Chem. Engineering Journal*, **2017**, *322*, 73–81.
7. Dewei Wang, Yatong Wang, Haiwei Liu, Wen Xu, Lang Xu. Unusual carbon nanomesh constructed by interconnected carbon nanocages for ionic liquid-based supercapacitor with superior rate capability. *Chem. Engineering Journal*, **2018**, *342*, 474–483.
8. Zhiwei Lu, Xiaochao Xu, Yujuan Chen, Xiaohui Wang, Li Sun, Kelei Zhuo. Nitrogen and sulfur co-doped graphene aerogel with hierarchically porous structure for high-performance supercapacitors. *Green Energy & Environment*, **2019**, doi:10.1016/j.gee.2019.06.001.
9. Qiu-hong Zhang, Song-lin Zuo, Xin-yu Wei, Yong-fangWang. H_3PO_4 activated carbons as the electrode materials of supercapacitors using an ionic liquid electrolyte. *Carbon*, **2018**, *134*, 537–537.
10. Yuqian Cui, Lulu Cheng, Changna Wen, Yutao Sang, Peizhi Guo, X.S. Zhao. Capacitive behavior of chestnut shell-based porous carbon electrode in ionic liquid electrolytes. *Colloids and Surfaces A: Physicochemical and Engineering Aspects*, **2016**, *508*, 173–177.
11. Ziyang Song, Dazhang Zhu, Liangchun Li, Tao Chen, Hui Duan, Zhiwei Wang, Yaokang Lv, Wei Xiong, Mingxian Liu, Lihua Gan. Ultrahigh energy density of a N, O codoped carbon nanosphere based all-solid-state symmetric supercapacitor. *J. Mater. Chem. A*, **2019**, *7*, 1177–1186.
12. J.-K.; Ewert, D.; Weingarth, C.; Denner, M.; Friedrich, M.; Zeiger, A.; Schreiber, N.; Jackel, V.; Presser, R. Kempe. Enhanced capacitance of nitrogen-doped hierarchically porous carbide-derived carbon in matched ionic liquids. *J. Mater. Chem. A*, **2015**, *3*, 18906–18912.
13. Haitao Zhang, Kai Wang, Xiong Zhang, He Lin, Xianzhong Sun, Chen Li, Yanwei Ma. Self-generating graphene and porous nanocarbon composites for capacitive energy storage. *J. Mater. Chem. A*, **2015**, *3*, 11277–11286.
14. Girum Ayalneh Tiruye, David Munoz-Torrero, Thomas Berthold, Jesus Palma, Markus Antonietti, Nina Fechler, Rebeca Marcilla. Functional porous carbon nanospheres from sustainable precursors for high performance supercapacitors. *J. Mater. Chem. A*, **2017**, *5*, 16263–16272.
15. C.; Zheng, X.F.; Zhou, H.L.; Cao, G.H.; Wang, Z.P.; Liu, Controllable synthesis of activated graphene and its application in supercapacitors. *J. Mater. Chem. A*, **2015**, *3*, 9543–9549.
16. Yongsheng Zhou, Pan Jin, Yatong Zhou, Yingchun Zhu. Carbon nanospheres hanging on carbon nanotubes: A hierarchical three-dimensional carbon nanostructure for high-performance supercapacitors. *J. Mater. Chem. A*, **2017**, *5*, 16595–16599.
17. Zhibin Lei, Zonghuai Liu, Huanjing Wang, Xiuxia Sun, Li Lu, X.S. Zhao. A high-energy-density supercapacitor with graphene–CMK-5 as the electrode and ionic liquid as the electrolyte. *J. Mater. Chem. A*, **2013**, *1*, 2313-2321.
